# Circular intermediate-mediated horizontal transfer of the chromosome-encoded *cfr(C)* gene in multi-drug resistant *Campylobacter coli* from swine sources

**DOI:** 10.3389/fmicb.2023.1274245

**Published:** 2023-12-22

**Authors:** Jae-Uk An, Soomin Lee, Jae-Ho Guk, Jungha Woo, Hyokeun Song, Seongbeom Cho

**Affiliations:** College of Veterinary Medicine and Research Institute for Veterinary Science, Seoul National University, Seoul, Republic of Korea

**Keywords:** *Campylobacter coli*, chromosome-encoded, circular intermediate, horizontal transfer, whole genome sequencing

## Abstract

*Campylobacter* is a major zoonotic pathogen that causes gastrointestinal and, rarely, immune diseases in humans. The antimicrobial-resistance gene *cfr(C)* carried by *Campylobacter* and is a *cfr*-like gene that targets bacterial 23S rRNA through A2503 methylation. *cfr(C)* confers cross-resistance to five antimicrobial classes (PhLOPS_A_), including lincosamide, streptogramin A, and pleuromutilin, which are classified as critically important antimicrobials to human by the World Health Organization. To elucidate the genetic variation and horizontal transfer mechanism of *cfr(C)*, we analyzed the genetic background and horizontal transfer unit of *Campylobacter*-derived *cfr(C)* through comparative genomic analysis. We identified nine *cfr(C)*-positive *C. coli* strains of 157 strains isolated from swine sources. Three novel *cfr(C)* gene single nucleotide polymorphism (SNP) sites (19delA, 674C > A, and 890 T > C) were identified from nine *cfr(C)*-positive strains. Among six identified *cfr(C)* SNP variant types (SNP-I to -VI), five types of randomly inserted *cfr(C)*-cassettes on chromosome and one type of plasmid-like element were identified, their gene cassette composition differing depending on the *cfr(C)* variants. Three of six *cfr(C)* cassette types contained aminoglycoside-streptothricin resistance cluster “*aphA3*-*sat4*-*aadE*.” The *cfr(C)* gene cassette with *pcp* gene (GC-1, GC-4, and GC-5) formed a *pcp*-mediated circular intermediate “*pcp*-hp-*cfr(C)*-*aphA3*,” which has not been previously reported. Other two *cfr(C)* cassette-types with ISChh1 formed circular intermediate “ISChh1-*aphA3*-*cfr(C)*-*lnu (G)*-*pnp*-*ant1*-hp-ATPase” and “ISChh1-*aphA3*-*cfr(C)*-hp.” In conjugation assay, the *pcp*-mediated circular intermediate was naturally transferred to the plasmid of recipient *C. coli* wild-type strain from swine source, and comparative genomic analysis revealed that *cfr(C)* encoded in *pcp*-mediated circular intermediate was inserted into the plasmid of recipient by homologous recombination with *pcp* and *aphA3*. This study revealed that novel multidrug resistance gene *cfr(C)* carried by *C. coli* from swine sources can be highly genetically diverse and transferable. Moreover, we suggest that the transferability of chromosomal *cfr(C)* may contribute to the global spread of multidrug resistance against clinically important antimicrobials.

## 1 Introduction

*Campylobacter* species, particularly *C. jejuni* and *C. coli*, are leading zoonotic foodborne pathogen that can cause gastrointestinal infections (such as diarrhea) and immune diseases (such as Guillain-Barré syndrome) in humans (Silva et al., 2011). Unlike in humans, *Campylobacter* causes mostly asymptomatic infections in food-animals, including poultry, cattle, and pigs, and is considered a commensal bacterium (Burnham and Hendrixson, 2018). According to the World Health Organization (WHO), the major route of human campylobacteriosis is via the food-chain of animal products (such as meat and milk) which are contaminated with *Campylobacter*, present in animal feces, during the slaughter process (Jaakkonen et al., 2020).

Since 2013, the US Centers for Disease Control and Prevention has classified drug-resistant *Campylobacter* as a serious antibiotic resistance threat (CDC, 2013). In recent years, *Campylobacter* has become increasingly resistant to clinically important antimicrobials and has developed multiple mechanisms of antimicrobial resistance. These include mutations in target genes, such as 23S rRNA mutations that confer resistance to macrolides and, *gyrA* mutations associated with fluoroquinolone resistance; multidrug efflux pump CmeABC/RE-CmeABC extruding structurally diverse compounds and antimicrobials; and horizontally acquired antimicrobial resistance genes, such as *tet(O)*, *erm(B)*, *fexA*, and *fosXCC* (Aarestrup et al., 2008). Among the two major species of *Campylobacter*, *C. jejuni* and *C. coli*, which are mainly responsible for human infection, *C. coli* is known to be more resistant to antimicrobials (Liu et al., 2019b).

The *cfr* gene, which encodes 23S rRNA methyltransferase, confers resistance to five antimicrobial classes, namely phenicols, lincosamides, oxazolidionones, pleuromutilins, and streptogramin A, known as PhLOPS_A_ phenotype (Schwarz et al., 2000; Long et al., 2006). WHO classified lincosamides, pleuromutilins, and streptogramin A as “critically important antimicrobials: highly important” and warned that resistance to these antimicrobial agents would make it difficult to treat bacterial infection (WHO, 2019). Since the first report of *cfr* gene in *Staphylococcus sciuri* in 2006, five types of the *cfr* gene family have been reported: *cfr*, *cfr(B)*, *cfr(C)*, *cfr(D)*, and *cfr(E)* (Schwarz et al., 2000; Deshpande et al., 2015; Hansen and Vester, 2015; Candela et al., 2017; Tang et al., 2017; Sassi et al., 2019; Stojković et al., 2019). Tang et al. (2017) first identified plasmid-mediated multi-drug resistance gene *cfr(C)* in *C. coli* isolates of feedlot cattle origin (Tang et al., 2017). Since the first report of *cfr(C)* in *Campylobacter*, three additional studies reported the presence of *cfr(C)* in *Campylobacter* species (Zhao et al., 2019; Liu et al., 2019a; Tang et al., 2020). However, the horizontal transferability of *Campylobacter*-derived *cfr(C)* has been reported in only one study (Tang et al., 2017), and the transfer mechanism of *cfr(C)* is still unclear and requires further research.

An understanding of genetic variation of *cfr(C)* and their transmission mechanisms can help control the propagation of antimicrobial multi-resistance, a serious problem when it is transferred to humans. Thus, in this study we analyzed genetic background and horizontal transfer unit of *cfr(C)* in *C. coli* strains through comparative genomic analysis to elucidate the genetic variation and horizontal transfer mechanism of the *Campylobacter*-derived *cfr(C)* gene from swine sources.

## 2 Materials and methods

### 2.1 Identification of *cfr(C)*-positive *Campylobacter coli* strains

A total of 157 swine-derived *C. coli* strains were used to screen *cfr(C)* presence. Of these, 130 *C. coli* strains were isolated from six swine farms in South Korea in our previous study (Guk et al., 2021). In addition, 15 *C. coli* strains were collected from 30 swine fecal samples from one slaughterhouse in South Korea in November 2017 and 12 *C. coli* strains from 30 fecal samples from one swine farm in South Korea in April 2018. Confirmation of *cfr(C)* was conducted using polymerase chain reaction (PCR). The primer used and annealing temperature are presented in Supplementary Table 1.

### 2.2 Antimicrobial susceptibility testing

Minimum inhibitory concentration (MIC) tests were conducted using Sensititre CAMPY2 plates (ThermoFisher Scientific, USA) following the manufacturer’s instructions for the following eight antimicrobial agents: azithromycin, ciprofloxacin, erythromycin, gentamicin, tetracycline, florfenicol, nalidixic acid, and clindamycin. The MIC value of each antimicrobial agent was interpreted according to the criteria of the National Antimicrobial Resistance Monitoring System for enteric bacteria.^1^ *C. jejuni* ATCC 33560 was used as the quality control strain for the MIC test.

### 2.3 Whole genome sequencing (WGS)

Total genomic DNA was extracted using NucleoSpin Microbial DNA kit (Macherey-Nagel, Germany) following the manufacturer’s instructions. Total genomic DNA was sequenced via a combination of NextSeq^®^ 500 technology (Illumina, Inc., USA) and MinION platforms (Oxford Nanopore Technologies, UK). Raw short-read and long-read data were assembled using hybrid-assembly strategy in Unicycler (v0.5.0) (Wick et al., 2017) and annotated using prokka (v1.14.5) (Seemann, 2014).

### 2.4 Identification of novel *cfr(C)* variants via single nucleotide polymorphism (SNP) analysis

To identify *cfr(C)* variant types, the nucleotide sequences of *cfr(C)* of nine isolated *C. coli* strains were aligned with that of the reference strain, *C. coli* Tx40 (National Center for Biotechnology Information [NCBI] accession number: NG_060579). Thereafter, we conducted *cfr(C)* gene SNP-based comparative genomic analysis by comparing the *cfr(C)* sequences of nine strains with that of 28 *C. coli* strains, the WGS of which were deposited in the NCBI database. In addition, SNP-based phylogenetic analysis was performed for *cfr(C)* of nine *cfr(C)*-positive strains together with that of other strains published in NCBI database. Phylogenetic tree was constructed with MEGAX software by using UPGMA method (Kumar et al., 2018). Bootstrap values were calculated with 1,000 replications. The accession numbers of nucleotide sequences are listed in Supplementary Table 2.

### 2.5 Functional confirmation of *cfr(C)* variants through cloning experiments

To determine the role of *cfr(C)* in conferring florfenicol resistance, a 1,425 bp DNA fragment was amplified from eight *C. coli* strains (CC021, 027, 032, 041, 042, 046, 135, and 159) through PCR. This fragment included the coding sequence of *cfr(C)* (1,140 bp) as well as 63 bp upstream and 163 bp downstream sequences of the gene. For *C. coli* strain CC024, a 1,327 bp DNA fragment was amplified through PCR. This fragment included the coding sequence of *cfr(C)* as well as the 122 bp upstream and 163 bp downstream sequences of the gene. The amplicon was ligated with an *E. coli*/*C. jejuni* shuttle plasmid pUC19 (Yanisch-Perron et al., 1985) to construct plasmid pUC19-*cfr(C)*. The pUC19-*cfr(C)* plasmid was then transformed into *E. coli* DH5α competent cells by heat shock transformation. The *E. coli* transformants were selected from Luria-Bertani agar (Sigma-Aldrich, USA) plates containing 50 mg/L ampicillin and 50 mg/L kanamycin. The confirmation for carrying plasmid pUC19-*cfr(C)* was performed thorough PCR using the *cfr(C)*-specific primers. MIC tests were also conducted for *E. coli* transformants to evaluate the antimicrobial resistance. Used primer and annealing temperature used in the experiments are listed in Supplementary Table 2.

### 2.6 Analysis of *cfr(C)*-carrying gene cassettes and circular intermediates

The *cfr(C)*-carrying gene cassettes of nine *C. coli* strains were aligned against the corresponding region of reference strain *C. coli* Tx40 ([NCBI] accession number: NG_060579) and *C. coli* SHP40 ([NCBI] accession number: MF037584). Additionally, the inserted positions of *cfr(C)*-carrying gene cassette in nine *C. coli* strains were identified by alignment and comparison with *C. coli* strain ATCC33559. The comparative genomic visualization was performed using “Easyfig (v2.2.3)” (Sullivan et al., 2011). The circular intermediate form of *cfr(C)*-carrying gene cassette was investigated by using inverse PCR and Sanger sequencing. Amplicon sequences were aligned and compared with sequences submitted in the NCBI GenBank database using the Basic Local Alignment Search Tool program (BLAST).^2^ The primers and annealing temperature used in the experiments are listed in Supplementary Table 2.

### 2.7 Conjugation assay

To evaluate the horizontal transferability of *cfr(C)*-carrying gene cassette, we conducted conjugation assay following the previously described methods with slight modification (Davis et al., 2008). The conjugation assay was performed with a gentamicin-resistant wild-type *C. coli* as the recipient and eight *cfr(C)*-positive *C. coli* strains as the donors: CC021, CC027, CC032, CC041, CC042, CC046, CC135, and CC159. Among nine *cfr(C)*-positive strains included in this study, CC024 was excluded from the conjugation assay with the gentamicin-resistant recipient strain, since it carried resistance to gentamicin. To select the transconjugants, Mueller-Hinton agar (Sigma Aldrich) plates containing 1 mg/L gentamicin and 4 mg/L florfenicol were used. The presence of *cfr(C)* gene and circular intermediate form in transconjugants were confirmed using PCR. The antimicrobial resistance was evaluated for transconjugants though MIC tests.

## 3 Results

### 3.1 Novel *cfr(C)* SNP variants

**Figure 1:**
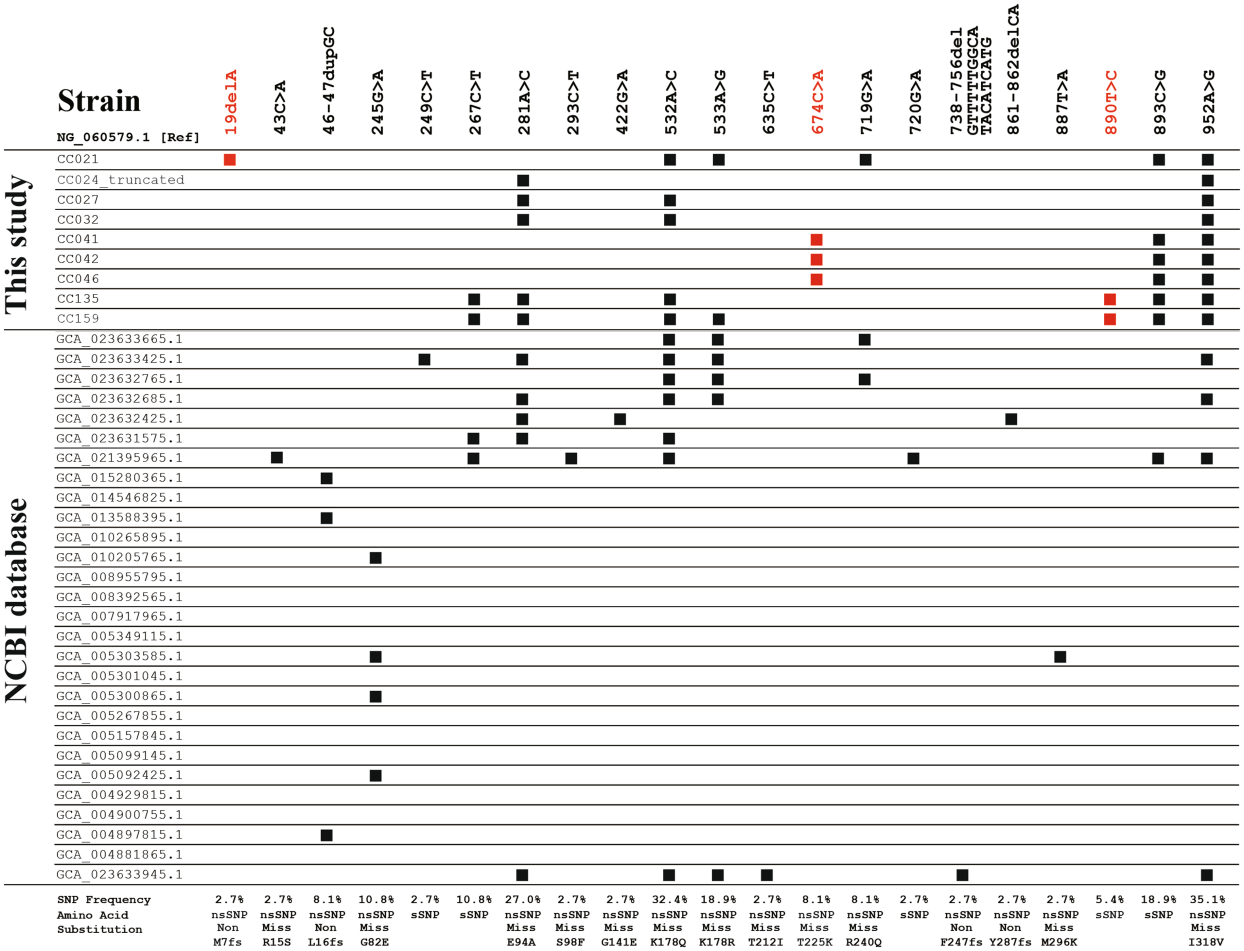
Single nucleotide polymorphic (SNP) sites of *cfr(C)* in 37 strains, including nine strains identified in this study and 28 strains obtained from NCBI database. Nucleotide sequence of *cfr(C)* (1,140 bp) of *C. coli* Tx40 strain (NG_060579.1) was used as reference. SNP sites shown in red font represent the novel SNP sites identified in this study. nsSNP, non-synonymous SNP; sSNP, synonymous SNP; Non, non-sense nsSNP; Miss, Miss-sense nsSNP; fs, frameshift mutation.

Among the 157 swine-derived *C. coli* strains, nine *cfr(C)*-positive strains were identified (Table 1). A total of six SNP variant types were identified in SNP-based comparative genomic analysis: SNP-I (CC021), SNP-II (CC024), SNP-III (CC027 and CC032), SNP-IV (CC041, CC042, and CC046), SNP-V (CC135), and SNP-VI (CC159) (Figure 1 and Supplementary Figure 1). SNP-I carried six SNPs, including a nonsense SNP (19delA, M7fs), which caused frameshift mutation of *cfr(C)* in strain CC021. SNP-II carried two SNPs, 281A > C (E94A) and 952A > G (I318V), and this type was truncated at the 3′ end (1,101 bp). SNP-III carried three SNPs, 281A > C, 532A > C (K178Q), and 952A > G. The SNP-IV type carried three SNPs, including the novel SNP 674C > A (T225K), which was not identified in previously reported strains uploaded in the NCBI database. The SNP-V type carried six SNPs, including the novel SNP 890T > C (sSNP), which was not identified in previously reported strains uploaded in the NCBI database. The SNP-VI type carried seven SNPs, including 533A > G (K178R) and the novel SNP 890T > C.

**Table 1:** Descriptive information of *C. coli* strains used in this study.

	Farm	Stage	MLST (ST)	SNP profile type	FFN	CLI	Gene cassette	Circular intermediate	Conjugation	SNP
CC021	A	Growing	887	SNP-I	S	R	GC-1	CIR-1	Conjugated	19delA*^†^, 532A > C, 533A > G, 719G > A, 893C > G, 952A > G
CC024	B	Weaning	1,142	SNP-II	R	R	GC-2	-	-	281A > C, 952A > G
CC027	B	Growing	2,699	SNP-III	S	R	GC-3	CIR-2	Not conjugated	281A > C, 532A > C, 952A > G
CC032	B	Finishing	2,699	SNP-III	S	R	GC-3	CIR-2	Not conjugated	281A > C, 532A > C, 952A > G
CC041	C	Growing	830	SNP-IV	R	R	GC-4	CIR-1	Conjugated	674C > A*, 893C > G, 952A > G
CC042	C	Growing	830	SNP-IV	R	R	GC-4	CIR-1	Conjugated	674C > A*, 893C > G, 952A > G
CC046	C	Growing	830	SNP-IV	R	R	GC-4	CIR-1	Conjugated	674C > A*, 893C > G, 952A > G
CC135	D	Diseased	1,096	SNP-V	R	R	GC-5	CIR-1	Conjugated	267C > T, 281A > C, 532A > C, 890 T > C*, 893C > G, 952A > G
CC159	S	Pork	1,556	SNP-VI	R	S	GC-6	CIR-3	Not conjugated	267C > T, 281A > C, 532A > C, 533A > G, 890 T > C*, 893C > G, 952A > G

*Novel SNP sites identified in this study.

^†^The SNP site in *cfr(C)* leading to premature stop codon.

MLST, Multi-locus sequence typing; SNP, single nucleotide polymorphism; FFN, florfenicol; CLI, clindamycin; S, susceptible; R, resistant; GC, gene cassette type; CIR, circular intermediate type.

In the SNP-based phylogenetic tree, the *cfr(C)* nucleotide sequences of the nine strains were genetically similar to those of swine- and human-derived *C. coli* strains isolated from China rather than that of cattle, chicken, and human-derived *C. coli* strains isolated from USA (Supplementary Figure 2). In addition, the *cfr(C)* genes reported from the USA were genetically homogeneous regardless of region and year of isolation, whereas the *cfr(C)* genes reported from China showed high genetic variations.

### 3.2 Antimicrobial resistance of nine *cfr(C)*-positive *Campylobacter coli* strains and transformants

Two SNP types showed susceptibility to florfenicol: SNP-I (CC021) and SNP-III (CC027 and 032), with MIC values of 2 and 4 mg/L, respectively (Supplementary Table 3). The other types showed resistance to florfenicol (MIC: 8 mg/L). Resistance to clindamycin was identified in three SNP types: SNP-II, -III, and -V. Regardless of the SNP variants, strains CC024, CC027, CC032 and CC135 exhibited resistance to the macrolide class, namely azithromycin (MIC: ≥64 mg/L) and erythromycin (MIC: ≥64 mg/L). Except for the CC024 strain, all other types showed susceptibility to gentamicin (MIC: ≤4 mg/L). All strains except for CC159 strain showed resistance to the quinolone class, including ciprofloxacin (MIC: ≥1 mg/L) and nalidixic acid (MIC: ≥32 mg/L).

All six SNP types of *cfr(C)* were transformed into *E. coli* DH5α competent cells. The transformant of SNP-I type (CC021) exhibited susceptibility to florfenicol (MIC: 2 mg/L). The other SNP types (SNPs II–VI) exhibited resistance to florfenicol (MICs: 4–8 mg/L). For the clindamycin-resistant phenotype, MICs increased at least two-fold compared to that of *E. coli* DH5α competent cells (increase to ≥32 mg/L from 16 mg/L).

### 3.3 Gene-cassette carrying *cfr(C)*

**Figure 2:**
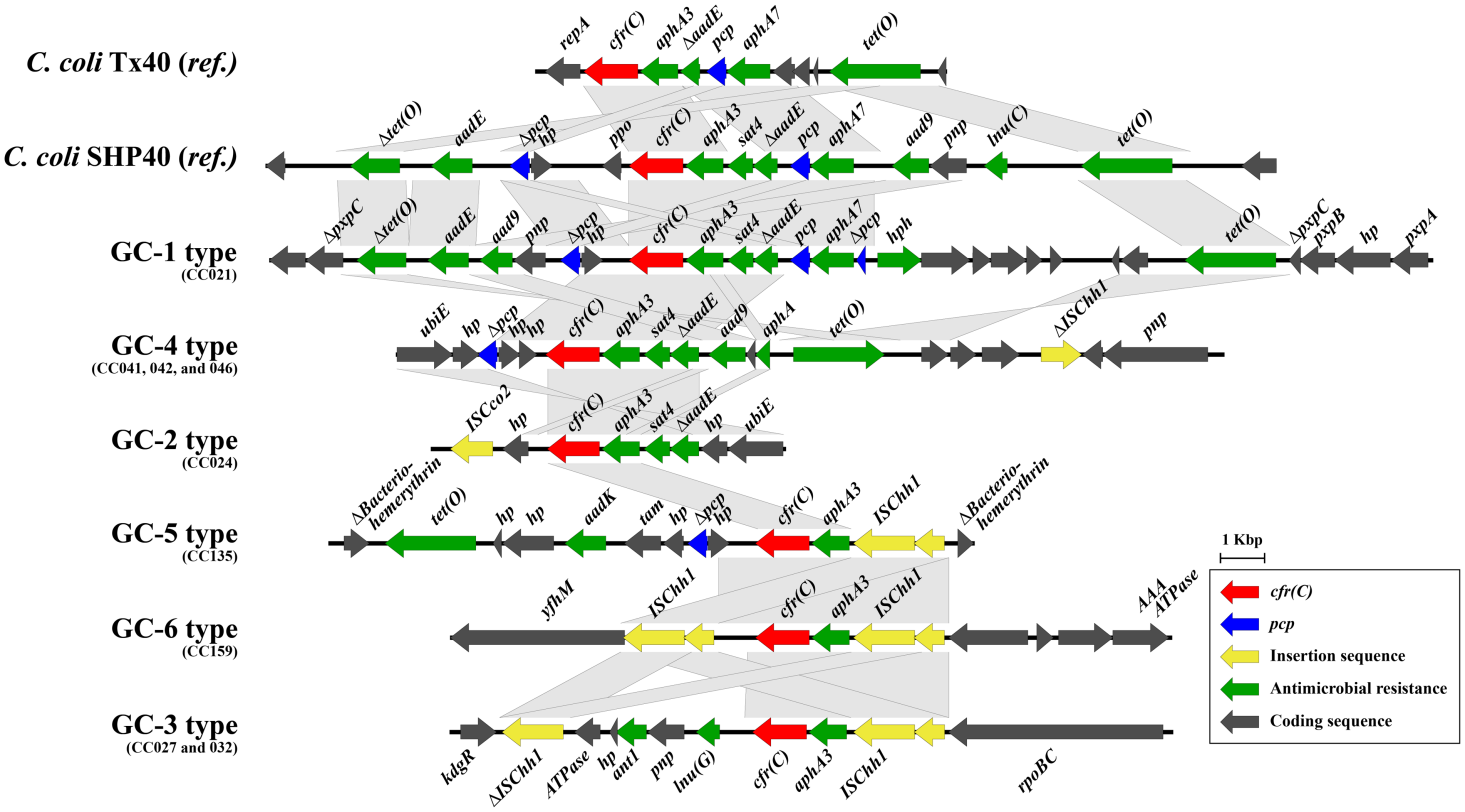
Six types of gene cassettes carrying *cfr(C)* identified in comparative genomic analysis of nine strains. The nucleotide sequences of *cfr(C)*-carrying gene cassettes were aligned and compared by using Easyfig (v2.2.3).

A total of six *cfr(C)*-carrying gene cassette types (GC-1–6) were identified, differing based on SNP-types (Figure 2 and Table 1). Except for the gene cassette of strain CC024, all five gene cassettes carrying *cfr(C)* were identified to be encoded on the chromosome. Of the 6 GC types, three types (GC-1, −4, and −5) were flanked by the *pcp* gene in the upstream of *cfr(C)*. Two types (GC-3 and -6) were bracketed by two copies of the IS607 family member ISChh1 upstream and downstream of *cfr(C)*. GC-5 was bracketed by one ISChh1 upstream of *cfr(C)* and downstream of *pcp*. In all six GC types, *aphA3* was encoded downstream of *cfr(C)*. The aminoglycoside-streptothricin resistance cluster “*aphA3*-*sat4*-Δ*aadE*” was identified from 3 GC types (GC-1, -2, and -4). The GC-1 type (CC021) was “Δ*pcp*-hp-*cfr(C)*-*aphA3*-*sat4*-Δ*aadE*-*pcp*,” in which the *ppo* gene was deleted compared to corresponding region of the reference *C. coli* SHP40 strain. The GC-4 type (CC041, 042, and 046) was “Δ*pcp*-hp-hp-*cfr(C)*-*aphA3*-*sat4*-Δ*aadE*,” with *pcp* deleted upstream of Δ*aadE*. The GC-2 type (CC024) was “hp-Δ*cfr(C)*-*aphA3*-*sat4*-Δ*aadE-*hp,” with *pcp* and hp. deleted in the gene cassette compared to the corresponding region of the GC-1 type. The GC-5 type (CC135) was “Δ*pcp*-hp-*cfr(C)*-*aphA3*-ISChh1.” The GC-6 type (CC159) was “ISChh1-*cfr(C)*-*aphA3*-ISChh1.” The gene cassette of the SNP-III type (GC-3) consisted of “ΔISChh1-ATPase-hp-*ant1*-*pnp (deoD)*-*lnu (G)*-*cfr(C)*-*aphA3*-ISChh1” and contained the insert ATPase-hp-*ant1*-*pnp*-*lnu(G)* compared with the corresponding region of GC-6.

### 3.4 Circular intermediate form of *cfr(C)*-carrying gene cassettes and horizontal transferability

**Figure 3:**
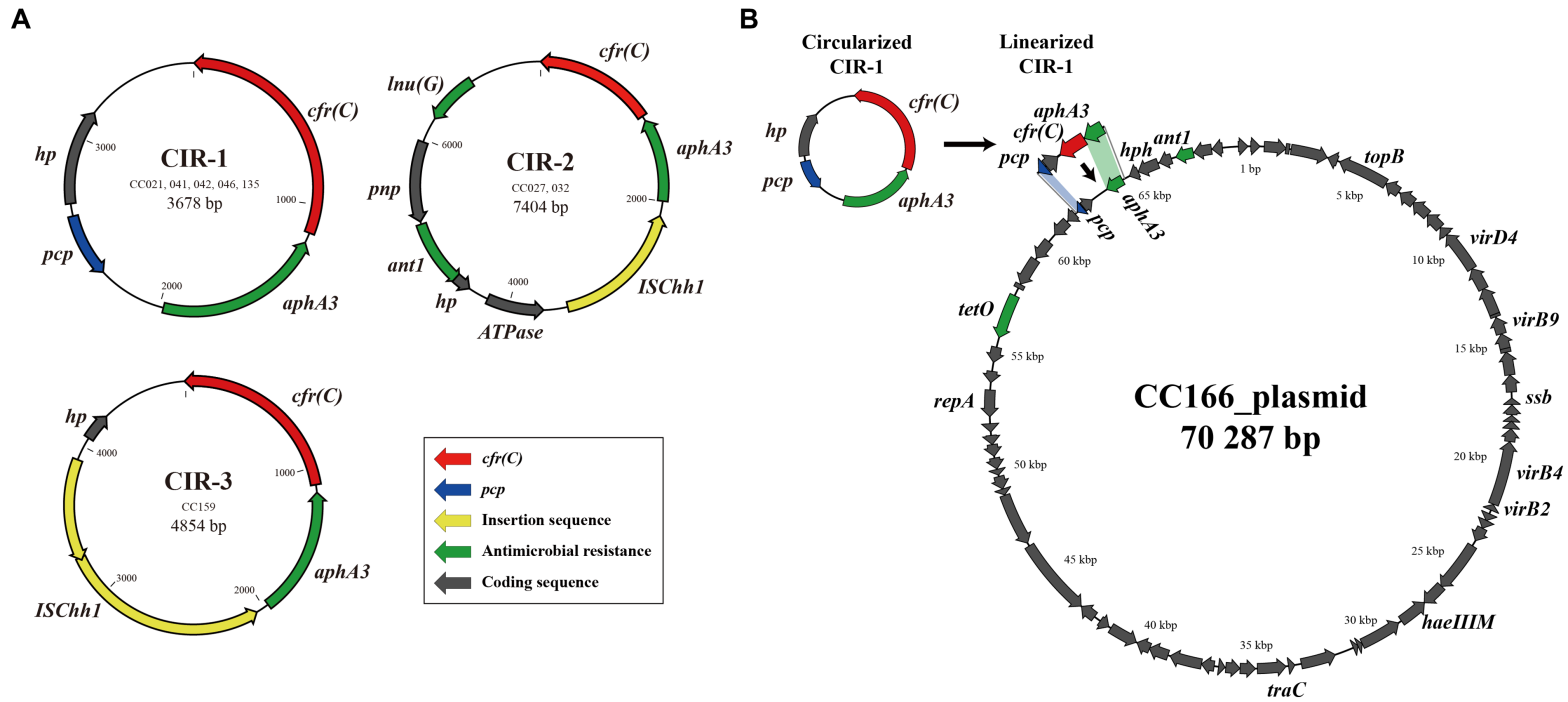
*cfr(C)*-carrying circular intermediates identified by inverse PCR and amplicon sequencing. Three types of circular intermediates were identified from *cfr(C)*-carrying *C. coli*, except for strain CC024. **(A)** Three types of circular intermediates formed by eight *C. coli* strains, **(B)** the genetic environment of recipient strain *C. coli* CC166 plasmid and insertion site of CIR-1.

Of the six GC types identified in this study, five formed circular intermediates of the *cfr(C)*-cassette. In total, three types of circular intermediates (CIR-1, CIR-2, and CIR-3) were identified (Figure 3A). The CIR-1 type, which was 3,678-bp long and consisted of “*pcp*-*hp*-*cfr(C)*-*aphA3*,” was identified from three GC types, GC-1, -4, and -5. The CIR-2 type, which was 7,833-bp long and consisted of “ΔISChh1-*aphA3*-*cfr(C)*-*lnu(G)*-*pnp*-*ant1*-hp-ATPase,” was identified from GC-3. CIR-3, which was 4,854-bp long and consisted of ‘“ISChh1-*aphA3*-*cfr(C)*-hp,” was identified from GC-6. The circular intermediate form of GC-2 was not identified.

In the conjugation assay, CIR-1 was confirmed to be horizontally transferred to recipient strain. The horizontal transferability of other two types, CIR-2 and CIR-3, was not identified. The transconjugants of CIR-1 exhibited acquired resistance to florfenicol (MIC: 8 mg/L) and clindamycin (MIC: 8 mg/L). In WGS analysis, the “*pcp*-hp-*aphA3”* region encoded on the plasmid of wild-type strain CC166 was replaced into “*pcp*-hp-*cfr(C)*-*aphA3”* after conjugation with a CIR-1 type strain (Figure 3B). As a result, the size of the plasmid increased from 70,287 to 71,195 bp.

### 3.5 Favored insertion site of *cfr(C)*-carrying gene cassette

**Figure 4:**
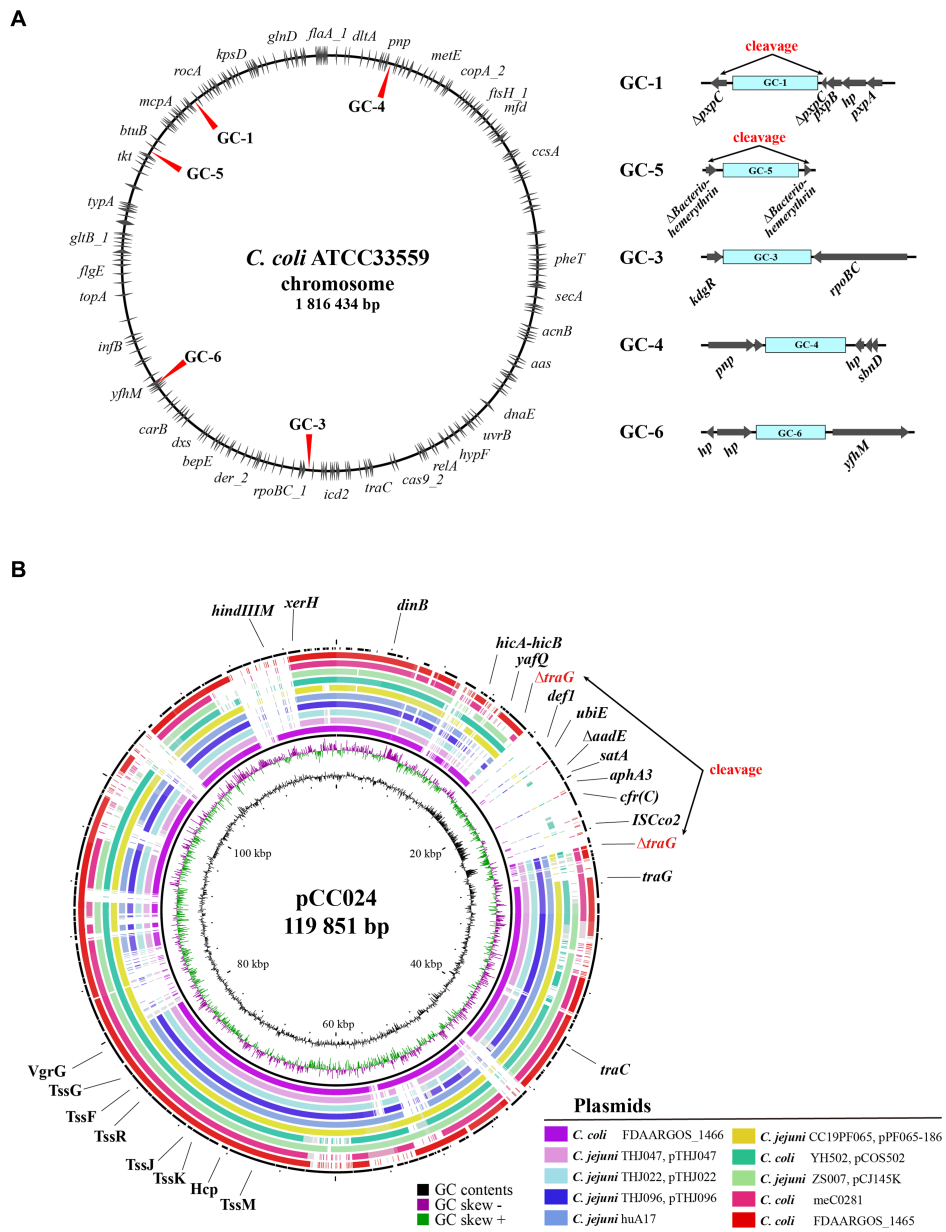
Insertion sites of *cfr(C)*-carrying gene cassette in wild type *cfr(C)*-carrying *C. coli*. **(A)** Chromosomal insertion sites of each *cfr(C)*-carrying gene cassette types based on nucleotide sequence of *C. coli* strain ATCC33559. **(B)** Comparative genomic analysis of *cfr(C)*-carrying plasmid-like genetic element of CC024 strain by using BRIG. For comparison, nucleotide sequences of 10 plasmids of *Campylobacter* species with high genetic homology to the plasmid of CC024 were obtained from NCBI database by using BLAST.

The genomic island of the GC-1 type was composed of Δ*tetO*-*aadE*-*aad9*-*pnp*-Δ*pcp*-hp-*cfr(C)*-*aphA3*-*satA*-Δ*aadE*-Δ*pcp*-*hph*-hp (2)-*traC*-hp (6)-*tetO*, which was inserted into the *pxpC* gene of the *pxpABC* complex at positions 249,516–268,882 bp (19,367 bp), based on the location of the *dnaA* gene that encodes the chromosomal replication protein (Figure 4 and Supplementary Figure 3). GC-4, which was part of a genomic island composed of ΔISChh1-hp (3)-*tetO*-*aphA*-hp-*aad9*-hp-*sat4*-*aphA3*-*cfr(C)*-hp (2)-Δ*pcp*-hp-*ubiE* (14,424 bp), was inserted at positions 1,184,369–1,198,793 bp encoding *lptD*-*pnp*-hp upstream and hp (2)-*sbnD* downstream. For GC-5, the *cfr(C)*-carrying genomic island (11,787 bp), which was composed of ISChh1-*aphA3*-*cfr(C)*-hp (2)-*tam*-*aadK*-hp (2)-*tetO*, was inserted at positions 165,574–177,361 bp on the gene encoding bacteriohemerythrin. As a result of this insertion, both sides of the GC-5 genomic island’s insertion site encoded a truncated bacteriohemerythrin gene. The GC-6 genomic island consisted of ISChh1-*aphA3*-*cfr(C)*-ISChh1 and was found to be inserted at positions 227,744–234,515 bp, flanked by “*purE*-*dapB*-AAA ATPase-hp (3)” and “*yfhM*-*mtgA*-hp” on each side. The GC-3 type was composed of ΔISChh1-ATPase-hp-*ant1*-*pnp*-*lnu(G)*-*cfr(C)*-*aphA3*-ISChh1, inserted at positions 1,261,026–1,270,356 bp and flanked by “*uxaA*-*dapA*-*kdgR*” and “*rpoBC*-*rplL*” on each side. Lastly, GC-2 of strain CC024 was identified within a circular genetic element with a size of 119,851 bp. The genomic island containing GC-2 was inserted into the *traG* gene.

## 4 Discussion

The rRNA methyltransferase N, encoded by *cfr* gene, causes m^8^-modification of A2503 of 23S rRNA, which is known to confer resistance to two antimicrobial agents, florfenicol and clindamycin (Long et al., 2006). Even though *cfr(C)* is known to induce antimicrobial resistance to critically important antimicrobials to humans, its transfer mechanism is still unclear and requires further research. In this study, nine novel *cfr(C)*-positive *C. coli* strains were isolated from swine sources (swine farms and slaughterhouses). To elucidate the horizontal transfer mechanism of the *Campylobacter*-derived *cfr(C)* gene, we analyzed the genetic background and horizontal transfer unit of *cfr(C)* in *C. coli* strains through comparative genomic analysis.

Compared to the previously reported sequences of *cfr(C)*, two strains (CC021 and CC024) carried incomplete amino acid coding sequences of the *cfr(C)* gene. Insertion or deletion of one or two adenines at the polyadenine site, such as 19delA (amino acid: M7fs) identified in CC021, generates premature stop codons by downstream frameshifting, leading to premature chain termination (Park et al., 2000). Tang et al. (2020) reported loss of resistance to florfenicol and clindamycin of *cfr(C)* due to other nucleotide deletion mutations in the *C. coli* JP10 strain, in which a frameshift mutation occurred due to nucleotide deletion at position 738–756 of *cfr(C)*. The *cfr(C)* amino acid sequence of CC024 (366 amino acids) exhibited 95.51% identity to that of *C. coli* Tx40 (379 amino acids). Notably, the original CC024 strain, as well as its transformant strain, showed resistance to florfenicol. According to a study of *cfr* protein structure and function, the function of the *cfr* protein analog was ensured by a highly conserved amino acid sequence around the C-terminus (G.DIdAACGQL) (Atkinson et al., 2013). In this study, although the amino acid sequence of the *cfr(C)* gene was truncated in the CC024 strain, the conserved C-terminal region of *cfr(C)* was preserved, which may ensure the function of *cfr(C)*.

Remarkably, the transformants of CC027 and CC032 with three missense non-synonymous (ns) SNPs (281A > C, 532A > C, and 952A > G) showed weak resistance to florfenicol (4–8 mg/L). These three missense nsSNPs were also identified in the florfenicol resistant *cfr(C)*-positive strains, CC135 and CC159. The amino acid substitutions (E94A, K178Q, and I318V), due to three SNP sites, have also been identified in the florfenicol and clindamycin-resistant *C. coli* JZ_1_79 and SH96 strains reported by Tang et al. (2020). The *cfr(C)*-encoded rRNA methyltransferase has functional amino acid sites including three substrate entrance sites and eight substrate modulation sites (Atkinson et al., 2013). Considering that the mutation sites of CC027 and CC032 do not overlap with the mutation sites of the previous report, it can be inferred that the three SNPs of these two strains are not associated with the phenotype for florfenicol resistance. Therefore, in relation to decreased resistance to phenicols found in strains CC027 and CC032, further research on the genetic background and expression of *cfr(C)* is needed.

Except for CC159, all eight *cfr(C)*-positive *C. coli* strains showed resistance to clindamycin, although the resistance level was different between strains depending on the carriage of 23S rRNA A2075G substitution: four strains with 23S rRNA A2075G substitutions showed high-level clindamycin resistance, whereas strains without the 23S rRNA A2075G substitution showed low-level resistance. The 23S rRNA A2075G substitution is known to be the most common mechanism underlying high-level resistance to the macrolide and lincosamide classes of antimicrobial agents in *Campylobacter* (Perez-Boto et al., 2010). Considering that the resistance of bacteria to lincosamide is generally mediated by the combination of three mechanisms (ribosomal target methylation or mutation, efflux of antimicrobials, and drug inactivation) (Leclercq, 2002), strains with simultaneous *cfr(C)*-induced A2503 methylation and A2075G substitution could exhibit high resistance to the lincosamide class. Our result suggests that *cfr(C)*-induced A2503 methylation may lead to low-level resistance to clindamycin, which could be enhanced by the acquisition of A2075G substitution.

*cfr(C)* is encoded on transferable plasmids (Tang et al., 2017;Liu et al., 2019a; Tang et al., 2020) or the chromosome of *cfr(C)*-positive *C. coli* strains (Liu et al., 2019a; Tang et al., 2020). In this study, the *cfr(C)* gene was encoded on the bacterial chromosome in the form of gene cassettes in eight *cfr(C)*-positive *C. coli* strains, except the gene cassette of strain CC024, and a total of six different gene cassettes were identified. Consistent with previous studies, all six *cfr(C)* gene cassettes carried the aminoglycoside resistance gene, *aphA3*, which was encoded upstream of *cfr(C)* in the same direction (Tang et al., 2017; Zhao et al., 2019; Liu et al., 2019a; Tang et al., 2020). Three cassette types, namely GC-1 (CC021), GC-2 (CC041, 042, and 046), and GC-5 (CC024), carried the aminoglycoside-streptothricin resistance gene cluster “*aphA3*-*sat4*-*aadE*” in the upstream of the *cfr(C)*-cassette gene. The “*aphA3*-*sat4*-*aadE*” cluster was also reported to be encoded in the upstream of the *cfr(C)*-cassette in the *C. coli* SHP40 strain, where the *cfr(C)* gene was first reported on the chromosome (Liu et al., 2019a). In contrast, the aminoglycoside resistance gene cluster “*aphA3*-*aadE*” without “*sat4*” was encoded in the upstream of the *cfr(C)*-cassette in the plasmids of 2 *C. coli* strains, Tx40 and N61925F (Tang et al., 2017; Zhao et al., 2019). The *aphA3* gene is an aminoglycoside-resistance determinant, encoding aminoglycoside 3′-phosphotransferase, and the *sat4* gene is a streptothricin-resistance determinant, encoding streptothricin N-acetyltransferase (Lysnyansky and Borovok, 2021b). The “*aphA3*-*sat4*-*aadE*” cluster is one of the highly transmissible and disseminated clusters, mainly found in the plasmids of gram-positive (*Staphylococcus* and *Enterococcus*) and gram-negative bacteria (*Campylobacter*) (Boerlin et al., 2001; Lysnyansky and Borovok, 2021a; Guirado et al., 2022). The *aphA3*-*sat4*-*aadE* cluster is transmitted with various antimicrobial-resistance genes, such as *ermB*, *optrA*, and *tet (O)* (Werner et al., 2001, 2003; Palmieri et al., 2012). Although the *aphA3*-*sat4*-*aadE* cluster has been reported to be mediated by mobile genetic elements, such as Tn5405 (Werner et al., 2001), recent studies have also shown that in *Campylobacter* spp., this resistance gene cluster may be mediated by a process of homologous recombination (Qin et al., 2012). Homologous recombination may enable horizontal transfer and chromosomal integration of gene clusters without mobile genetic elements (de Vries and Wackernagel, 2002). In the case of GC-4, another resistance gene cluster, “*ant1*-*pnp*-*lnu(G)*,” was encoded in the upstream of the *cfr(C)*-cassette. The lincosamide resistance gene, *lnu (E)*, is encoded in the upstream of the ISEnfa5-flanked *cfr* gene cassette in the *Streptococcus suis* RN4220 strain (Zhao et al., 2014).

The random insertion of a gene cassette into *cfr(C)* has the potential to significantly affect the fitness and survival of *Campylobacter*, as the insertion of the *cfr(C)* gene cluster may interfere with crucial cellular processes of the bacterium. The insertion sites of GC-1 and -5, harboring genomic islands, were different from those of the *potD* gene reported by Tang et al. (2020). GC-1 was inserted into the *pxpC* gene, which encodes 5-oxoprolinase. 5-Oxoproline, a metabolite from the breakdown of glutamine, has been reported to inhibit bacterial growth when it accumulates without proper disposal (Niehaus et al., 2017). GC-5 was inserted into the bacteriohemerythrin-encoding gene. Hemerythrin helps maintain bacterial homeostasis through intracellular oxygen- and redox-sensing functions. Cells cannot rapidly adapt to environmental changes without hemerythrin (Kitanishi, 2022). Our results suggest that the cleavage of these two genes, caused by the insertion of *cfr(C)*-carrying GCs, could have detrimental effects on the survivability of *C. coli* strains. The GC-3, -4, and -6 types were inserted between genes without gene cleavage. The insertion site of GC-3 downstream of the *rpoBC* gene has been previously reported (Tang et al., 2020). However, the other two types of insertion sites, *pnp* to *sbnD* and ATPase-encoding gene to *yfhM*, are novel positions. In contrast, GC-2 (CC024) was inserted into a circular genetic element without a self-replication module, such as the replication initiation protein gene *repA*. This genetic element of CC024 was similar in genetic composition to the previously reported plasmids of *C. jejuni* and *C. coli*. In addition, differences in GC contents between insertion sites and other coding regions indicated that the *cfr(C)*-carrying cluster was not included in ancestral genetic elements. TraG, truncated by GC-2, plays major roles in conjugation, and is associated with the invasion of *C. jejuni* into epithelial cells (Poly et al., 2005). Thus, truncation of the *traG* gene could attenuate the pathogenicity and survivability of *Campylobacter*.

Of the six types of gene cassette, GC-1, -4, and -5 formed circular intermediates and were horizontally transferred to recipient *C. coli* strains. The transferred circular intermediate form was 3,678 bp in length and consisted of the four coding sequences “*pcp*-*hp*-*cfr(C)*-*aphA3.”* Although the present study found differences in the length and composition of *cfr(C)*-positive strains, the *pcp* gene-mediated circular intermediate “*pcp*-*hp*-*ppo*-*cfr(C)*-*aphA3*-*sat4*-*aadE*” has also been reported in the *C. coli* SHP40 strain (Liu et al., 2019a). Similar to our study, the *cfr(C)*-cassette was also encoded in the bacterial chromosome of the *C. coli* SHP40 strain. The *pcp* gene encodes an enzyme that is conserved in a variety of organisms, from prokaryotes (archaea and bacteria) to eukaryotes (Barrett et al., 2012). The *pcp* gene is found to be inserted at multiple sites in the bacterial genome, including chromosomes and plasmids, and this multiple insertion of *pcp* could mediate the intermolecular transfer of gene clusters by forming the circular intermediate: the circular intermediates released from chromosome/plasmids could be integrated into other chromosome/plasmids containing a homologous sequence, such as the *pcp* gene (Liu et al., 2019a). These characteristics of the *pcp* gene can facilitate the transmission of *cfr(C)*, which forms a cluster with *pcp*. Similarly, *tetO*-mediated circular intermediates of *ermB*, escaping from original plasmids/chromosome, could be integrated into other bacterial genomes by targeting the multiple inserted *tetO* homologous sequence (Liu et al., 2019b). In particular, the CC041, CC042, and CC046 strains that form GC-4 have been reported as hyper-aerotolerant strains, along with ST830, which is related to clinical isolates, according to our previous study (Guk et al., 2021). Since these strains not only transfer *cfr(C)* to other strains but also have a higher probability for transmission to humans than other *C. coli* strains, hyper-aerotolerant *cfr(C)*-positive *C. coli* potentially carry higher risk than other *cfr(C)*-positive *C. coli* strains.

In GC-3, 4, 5, and 6, *cfr(C)* was flanked by the IS607 family member ISChh1. The IS607 family is one of the major insertion sequences (ISs) that mediate transfer of antimicrobial resistance in *Campylobacter* spp. (Chen et al., 2018). For example, IS607 has been reported to mediate the transfer of the *optrA* gene, which induces resistance to oxazolidinones and phenicols, as well as of *fexA*, a florfenicol–chloramphenicol resistance gene (Tang et al., 2021). The IS607 family encoded in the upstream of the *cfr(C)*-gene cassette was also identified in the plasmids of the JZ_1_79, SH89, and JZ_1_74 strains reported by Tang et al. (2020). Among the three *cfr(C)-*cassette types flanked by IS607, GC-3 and GC-6 were bracketed by two copies of ISChh1 in the same orientation at both sides of the gene cassette and formed the ISChh1-mediated circular intermediates “ISChh1-*aphA3*-*cfr(C)*-*lnu(G)*-*pnp*-*antl1*-*hp*-ATPase” and “ISChh1-*aphA3*-*cfr(C)*-hp,” respectively. Even though the circular intermediates of GC-3 and -6, namely CIR-2 and -3, were not horizontally transferred in our study, two copies of IS elements in the same orientation can form a circular intermediate and can be transferred using the mechanism “copy-out-paste-in,” since the IS607 family is a highly active IS element (He et al., 2019). Tang et al. (2020) confirmed the existence of IS607 only upstream of the *cfr(C)*-cassette but could not confirm the formation of circular intermediates by the IS607 family. GC-4 and -5, bracketed by the ISChh1 segment and *pcp*, formed a circular intermediate mediated by *pcp* regardless of the position of the ISChh1 segment. These results suggest that two copies of IS607 in the same orientation play an important role in the formation of a circular intermediate form of gene cassettes.

The CIR-1s of five strains (CC021, 041, 042, 046, and 135) were horizontally transferred from the chromosomes into the plasmid of the recipient strain CC166 in the conjugation assay. For all five strains, the *pcp* and *aphA3* genes were present at the insertion sites, suggesting that insertion of CIR-1 may have been mediated by homologous recombination with *pcp* and *aphA3*. The conjugation of gene cassettes targeting homologous gene regions has been reported in several studies. Qin et al. (2012) suggested that the aminoglycoside resistance gene island of *C. coli* strain SX81 may be acquired from the *C. coli* strain RM2228 and mediated by homologous recombination of the *cadF* and *CCO1582* genes (Qin et al., 2012). Taken together with our results, this suggests that the use of *pcp*, which is a commonly encoded gene in *Campylobacter*, as a vector in the horizontal transfer of *cfr(C)*-carrying gene cassettes may be a possible reason for the global success of *cfr(C)* propagation.

This is the first study to elucidate the horizontal transferability of *cfr(C)* and random insertion site of the chromosome-encoded *cfr(C)* cassette in *Campylobacter* spp. The results reveal novel *cfr(C)* variants along with their associated genetic environments in *C. coli* isolates and indicate the flexibility of *C. coli* in acquiring new antimicrobial resistance genes. The transferability of chromosomal *cfr(C)* to humans may be attributed to the global spread of multidrug resistance against clinically important antimicrobials. Thus, enhanced surveillance is needed to monitor the emergence and spread of *cfr(C)* in *Campylobacter* from swine sources as well as other pathogens.

## Data availability statement

The datasets presented in this study can be found in online repositories. The names of the repository/repositories and accession number(s) can be found below: https://www.ncbi.nlm.nih.gov/, PRJNA1000181.

## Author contributions

J-UA: Conceptualization, Data curation, Formal analysis, Investigation, Methodology, Software, Validation, Visualization, Writing – original draft, Writing – review & editing. SL: Conceptualization, Data curation, Formal analysis, Investigation, Methodology, Resources, Software, Visualization, Writing – original draft, Writing – review & editing. J-HG: Conceptualization, Formal analysis, Methodology, Writing – original draft. JW: Conceptualization, Formal analysis, Methodology, Writing – original draft. HS: Writing – review & editing. SC: Conceptualization, Funding acquisition, Project administration, Supervision, Validation, Writing – review & editing.

## Footnotes

^1^https://www.cdc.gov/narms/antibiotics-tested.html

^2^https://blast.ncbi.nlm.nih.gov/Blast.cgi
