# Supplementary material for: Circular intermediate-mediated horizontal transfer of the chromosome-encoded cfr(C) gene in multi-drug resistant Campylobacter coli from swine sources
Source: Front Microbiol. 2023 Dec 22;14:1274245. doi: 10.3389/fmicb.2023.1274245 (PMC10770853; doi:10.3389/fmicb.2023.1274245)
Supplement: Supplementary file 1 [file Data_Sheet_1.pdf]

Supplementary Material

1 Supplementary Figures and Tables

1.1 Supplementary Figures

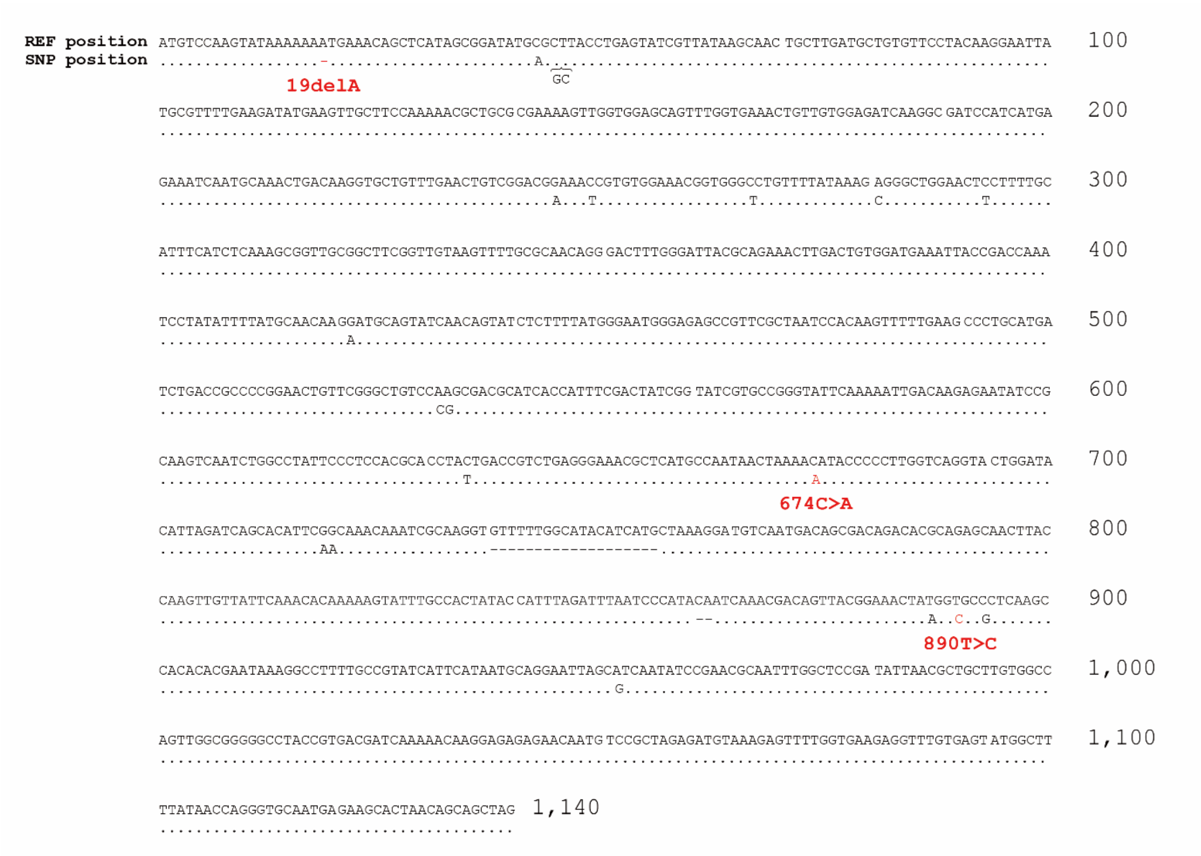

Supplementary Figure 1. Single nucleotide polymorphism (SNP) position of 37 *cfr(C)* variants, including nine strains identified in this study and 28 strains obtained from National Center for Biotechnology Information (NCBI) database. Nucleotide substitutions marked in red represent novel SNP sites identified in this study. Nucleotide sequence of *cfr(C)* (1140 bp) of *C. coli* Tx40 strain (NG_060579.1) was used to reference sequence position.

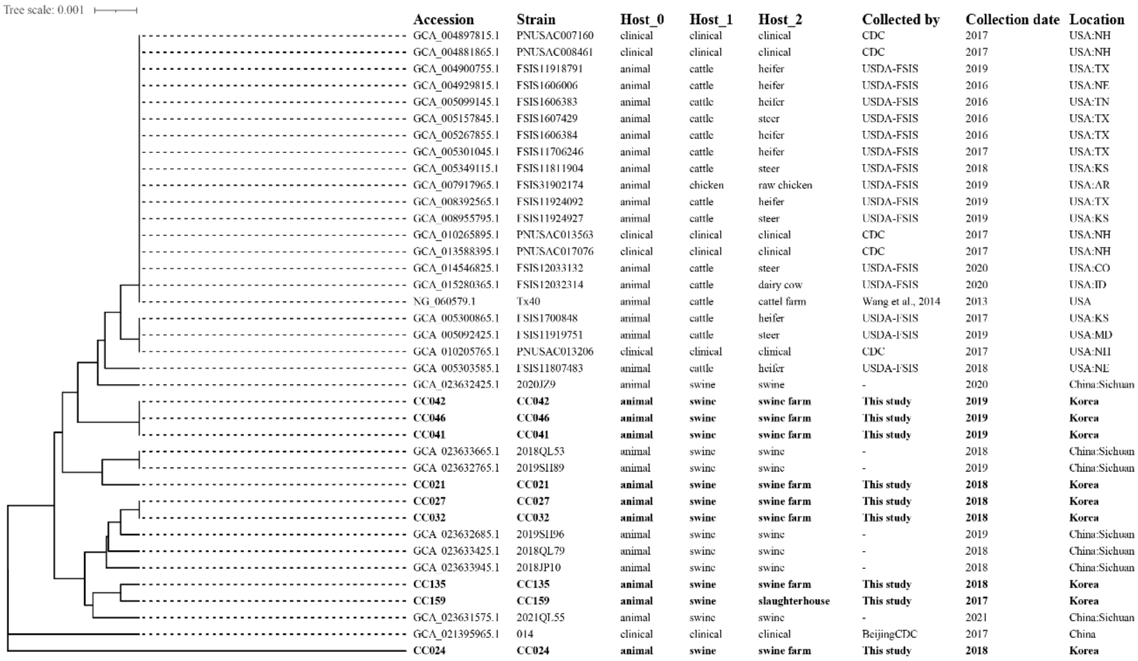

Accession	Strain	Host_0	Host_1	Host_2	Collected by	Collection date	Location
GCA_004897815.1	PNUSAC007160	clinical	clinical	clinical	CDC	2017	USA:NH
GCA_004881865.1	PNUSAC008461	clinical	clinical	clinical	CDC	2017	USA:NH
GCA_004900755.1	FSIS11918791	animal	cattle	heifer	USDA-FSIS	2019	USA:TX
GCA_004929815.1	FSIS1606006	animal	cattle	heifer	USDA-FSIS	2016	USA:NE
GCA_005099145.1	FSIS1606383	animal	cattle	heifer	USDA-FSIS	2016	USA:TN
GCA_005157845.1	FSIS1607429	animal	cattle	steer	USDA-FSIS	2016	USA:TX
GCA_005267855.1	FSIS1606384	animal	cattle	heifer	USDA-FSIS	2016	USA:TX
GCA_005301045.1	FSIS11706246	animal	cattle	heifer	USDA-FSIS	2017	USA:TX
GCA_005349115.1	FSIS11811904	animal	cattle	steer	USDA-FSIS	2018	USA:KS
GCA_007917965.1	FSIS31902174	animal	chicken	raw chicken	USDA-FSIS	2019	USA:AR
GCA_008392565.1	FSIS11924092	animal	cattle	heifer	USDA-FSIS	2019	USA:TX
GCA_008955795.1	FSIS11924927	animal	cattle	steer	USDA-FSIS	2019	USA:KS
GCA_010265895.1	PNUSAC013563	clinical	clinical	clinical	CDC	2017	USA:NH
GCA_013588395.1	PNUSAC017076	clinical	clinical	clinical	CDC	2017	USA:NH
GCA_014546825.1	FSIS12033132	animal	cattle	steer	USDA-FSIS	2020	USA:CO
GCA_015280365.1	FSIS12032314	animal	cattle	dairy cow	USDA-FSIS	2020	USA:ID
NG_060579.1	Tx40	animal	cattle	cattel farm	Wang et al., 2014	2013	USA
GCA_005300865.1	FSIS1700848	animal	cattle	heifer	USDA-FSIS	2017	USA:KS
GCA_005092425.1	FSIS11919751	animal	cattle	steer	USDA-FSIS	2019	USA:MD
GCA_010205765.1	PNUSAC013206	clinical	clinical	clinical	CDC	2017	USA:NH
GCA_005303585.1	FSIS11807483	animal	cattle	heifer	USDA-FSIS	2018	USA:NE
GCA_023632425.1	2020JZ9	animal	swine	swine	-	2020	China:Sichuan
**CC042**	**CC042**	**animal**	**swine**	**swine farm**	**This study**	**2019**	**Korea**
**CC046**	**CC046**	**animal**	**swine**	**swine farm**	**This study**	**2019**	**Korea**
**CC041**	**CC041**	**animal**	**swine**	**swine farm**	**This study**	**2019**	**Korea**
GCA_023633665.1	2018QL53	animal	swine	swine	-	2018	China:Sichuan
GCA_023632765.1	2019SH89	animal	swine	swine	-	2019	China:Sichuan
**CC021**	**CC021**	**animal**	**swine**	**swine farm**	**This study**	**2018**	**Korea**
**CC027**	**CC027**	**animal**	**swine**	**swine farm**	**This study**	**2018**	**Korea**
**CC032**	**CC032**	**animal**	**swine**	**swine farm**	**This study**	**2018**	**Korea**
GCA_023632685.1	2019SH96	animal	swine	swine	-	2019	China:Sichuan
GCA_023633425.1	2018QL79	animal	swine	swine	-	2018	China:Sichuan
GCA_023633945.1	2018JP10	animal	swine	swine	-	2018	China:Sichuan
**CC135**	**CC135**	**animal**	**swine**	**swine farm**	**This study**	**2018**	**Korea**
**CC159**	**CC159**	**animal**	**swine**	**slaughterhouse**	**This study**	**2017**	**Korea**
GCA_023631575.1	2021QL55	animal	swine	swine	-	2021	China:Sichuan
GCA_021395965.1	014	clinical	clinical	clinical	BeijingCDC	2017	China
**CC024**	**CC024**	**animal**	**swine**	**swine farm**	**This study**	**2018**	**Korea**

Supplementary Figure 2. Single nucleotide polymorphism (SNP) based phylogenetic analysis of 37 *cfr(C)* variants, including nine strains identified in this study and 28 strains obtained from National Center for Biotechnology Information (NCBI) database. Phylogenetic tree was constructed with MEGAX software by using UPGMA method. Bootstrap values were calculated with 1000 replications.

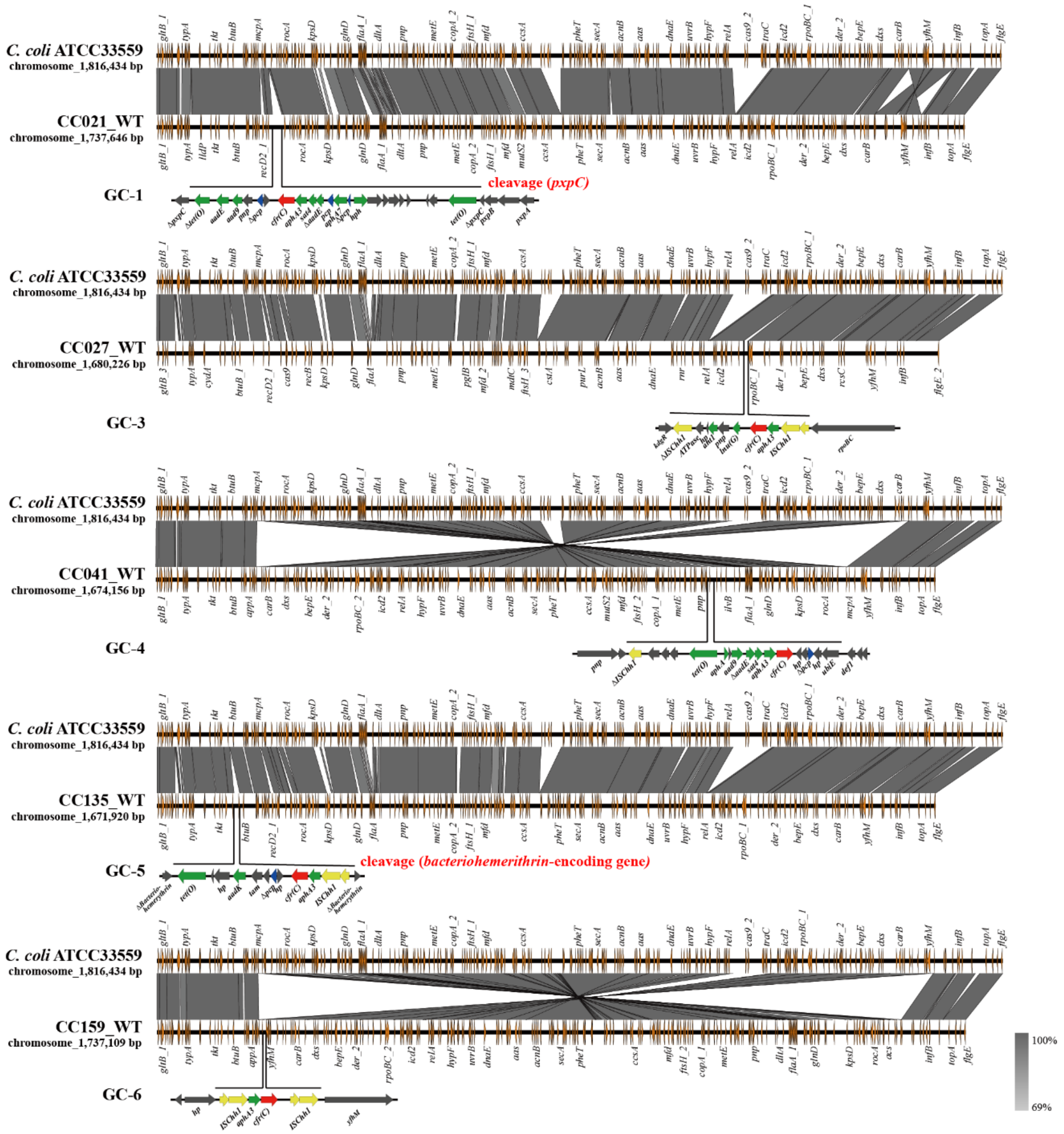

Supplementary Figure 3. Chromosome insertion sites of *cfr(C)*-carrying gene cassette in wild type *C. coli*. Chromosomal insertion sites of each gene cassette types based on comparative genomic analysis with *C. coli* strain ATCC33559 by using easyfig.

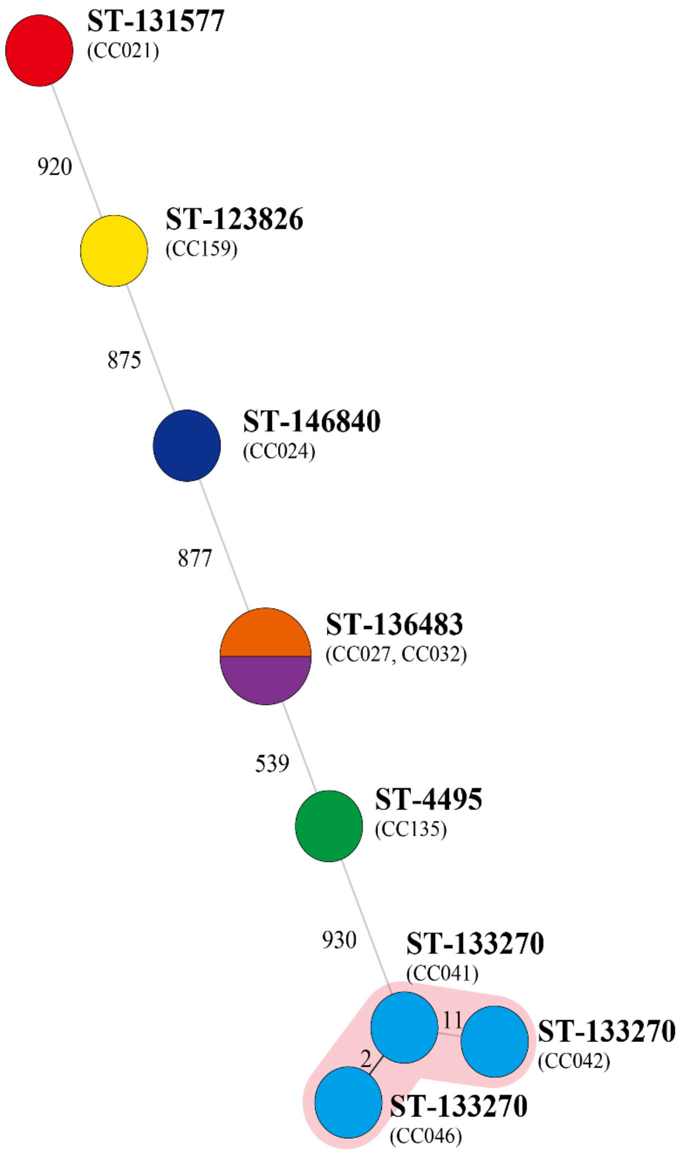

Supplementary Figure 4. Minimum spanning tree of nine *cfr(C)*-carrying *C. coli* based on Core genome multi-locus sequence typing (cgMLST). The closest matched core genome sequence types (ST) are represented by colored circles where size is proportional to the number of isolates. Each branch represents the number of an allelic differences between two linked STs.

1.2 Supplementary Tables

Supplementary table 1. Nucleotide sequences and annealing temperature of primers used in this study.

Target site		Nucleotide sequence (5′-3′)	Amplicon size (bp)	Annealing temperature (℃)	References
cfr(C)	F	GGTGAAACTGTTGTGGAGAT	722	60	Tang et al., 2017 [1]
	R	AGTTTCCGTAACTGTCGTTT			
cfr(C) inverse PCR	F	ACCAAACTGCTCCACCAAC	328	60	Liu et al., 2019 [2]
	R	TCCCATACAATCAAACGACA			
cfr(C) cloning	F (EcoRI)	CCGGAATTCATCAGGGAGGATATCGGG		60	This study
	R1 (XbaI)*	TGCTCTAGATTTTCGCAGCTGATAACGAG	1425		
	R2 (XbaI)†	TGCTCTAGAGAAAATTGCTCCCACATGGT	1317	60	

* *cfr(C)* cloning R1 (XbaI) primer was used for CC021, 027, 032, 041, 042, 046, 135, and 159 strains

† *cfr(C)* cloning R2 (XbaI) primer was used for CC024 strain

Supplementary table 2. National Center for Biotechnology Information (NCBI) accession numbers of 28 *cfr(C)* carrying *C. coli* strains for single nucleotide polymorphism (SNP) analysis.

**NCBI accession number of 28 strains used in the analysis of *cfr(C)* SNP variants**			
GCA_004881865.1	GCA_005301045.1	GCA_014546825.1	GCA_023632765.1
GCA_004897815.1	GCA_005349115.1	GCA_015280365.1	GCA_023633665.1
GCA_004900755.1	GCA_007917965.1	GCA_005099145.1	GCA_023633425.1
GCA_004929815.1	GCA_008392565.1	GCA_005300865.1	GCA_023632685.1
GCA_005092425.1	GCA_008955795.1	GCA_010205765.1	GCA_023633945.1
GCA_005157845.1	GCA_010265895.1	GCA_005303585.1	GCA_023631575.1
GCA_005267855.1	GCA_013588395.1	GCA_023632425.1	GCA_021395965.1

Supplementary table 3. Minimum inhibitory concentration of *cfr(C)*-carrying *C. coli* strains, transconjugants, and transformants

Strains	Minimum inhibitory concentration (mg/L)‡																Descriptions
	AZI		CIP		CLI		ERY		FFN		TET		GEN		NAL		
	S/R	MIC	S/R	MIC	S/R	MIC	S/R	MIC	S/R	MIC	S/R	MIC	S/R	MIC	S/R	MIC	
CC021	S	0.25	R	16	R	4	S	2	S	2	R	16	S	0.25	R	> 64	
CC024*	R	> 64	R	8	R	> 16	R	> 64	R	8	R	64	R	32	R	64	
CC027	R	> 64	R	8	R	> 16	R	> 64	S	4	R	> 64	S	0.25	R	> 64	
CC032	R	> 64	R	8	R	> 16	R	> 64	S	4	R	64	S	0.12	R	> 64	
CC041	S	0.25	R	16	R	2	S	2	R	8	R	> 64	S	0.12	R	> 64	Wild type *cfr(C)*-carrying *C. coli*
CC042	S	0.25	R	16	R	2	S	2	R	8	R	> 64	S	0.12	R	> 64	
CC046	S	0.25	R	16	R	2	S	4	R	8	R	> 64	S	0.12	R	> 64	
CC135	R	> 64	R	4	R	> 16	R	> 64	R	8	R	16	S	0.25	R	64	
CC159	S	0.06	S	0.12	S	1	S	0.5	R	16	R	4	S	0.12	S	4	
CC021_TC	S	0.12	R	16	R	8	S	1	R	8	R	64	R	> 32	R	64	
CC041_TC	S	0.06	R	8	R	8	S	1	R	8	R	32	R	32	R	64	
CC042_TC	S	0.06	R	8	R	8	S	1	R	8	R	32	R	32	R	64	Transconjugant
CC046_TC	S	0.06	R	16	R	8	S	1	R	8	R	> 64	R	> 32	R	64	
CC135_TC	S	0.06	R	16	R	8	S	1	R	8	R	64	R	32	R	64	
CC166	S	0.12	R	8	S	0.5	S	1	S	1	R	> 64	R	> 32	R	64	Recipient
DH5α/pUC19_CC021	R	2	R	0.03	R	> 32	R	64	S	2	S	2	S	0.12	R	64	
DH5α/pUC19_CC024	R	4	R	0.03	R	> 32	R	64	R	8	S	2	S	0.12	R	64	
DH5α/pUC19_CC027	R	2	R	0.03	R	> 32	R	64	R	4	S	2	S	0.12	R	64	
DH5α/pUC19_CC032	R	4	R	0.03	R	> 32	R	> 64	R	8	S	2	S	0.12	R	64	
DH5α/pUC19_CC041	R	4	R	0.03	R	> 32	R	64	R	8	S	1	S	0.12	R	64	Transformant with pUC19-*cfr(C)*
DH5α/pUC19_CC042	R	4	R	0.03	R	> 32	R	64	R	8	S	2	S	0.12	R	64	
DH5α/pUC19_CC046	R	4	R	0.03	R	> 32	R	64	R	8	S	2	S	0.12	R	64	
DH5α/pUC19_CC135	R	2	R	0.03	R	> 32	R	64	R	8	S	2	S	0.12	R	64	
DH5α/pUC19_CC159	R	4	R	0.03	R	> 32	R	> 64	S	4	S	2	S	0.12	R	64	
E. coli DH5α/pUC19†	R	4	R	0.03	R	16	R	64	S	4	S	1	S	0.12	R	64	Competent cell

* *cfr(C)* of CC024 was truncated (1101 bp)

† pUC19-vector with Amp^r, Kan^r

‡ AZI: azithromycin, CIP: ciprofloxacin, CLI: clindamycin, ERY: erythromycin, FFN: florfenicol, TET: tetracycline, GEN: gentamicin, NAL: nalidixic acid, R: resistant, S: Susceptible, TC: Transconjugant

2 Supplementary data

We conducted core genome multi-locus sequence typing (cgMLST) to evaluated the clonality of nine *cfr(C)*-carrying *C. coli* strains (Supplementary Figure 4). A total of 1343 loci from the "*C. jejuni*/*C. coli* cgMLST v1 scheme", provided by PubMLST, showed that CC041/CC042 and CC041/CC046 strains differed by only 11 and 2 loci, respectively. Additionally, the allele profiles of CC027 and CC032 strains were identical. These results support the possibility of their clonality. In the allele profile-based minimum spanning tree, the six identified sequence types (ST) based on cgMLST had between 539 to 930 allele differences observed between them. CC027/CC032 and CC041/CC042/CC046 are strains isolated from the same farm, and their clonality in cgMLST analysis are indicated that *cfr(C)*-carrying *C. coli* can be transmitted by clonal expansion of *C. coli* between swine at the same or different growth stages on the farm.
